# Measuring the Radius of Gyration and Intrinsic Flexibility of Viral Proteins in Buffer Solution Using Small-Angle X-ray Scattering

**DOI:** 10.1021/acsmeasuresciau.2c00048

**Published:** 2022-09-12

**Authors:** Riccardo Funari, Nikhil Bhalla, Luigi Gentile

**Affiliations:** † Department of Physics “M. Merlin”, University of Bari Aldo Moro, Via Amendola, 173, Bari 70125, Italy; ‡ Institute for Photonics and Nanotechnologies, CNR, Via Amendola, 173, Bari, 70125, Italy; § Nanotechnology and Integrated Bioengineering Centre (NIBEC), School of Engineering, Ulster University, Jordanstown, Shore Road, Northern Ireland BT37 0QB, United Kingdom; ∥ Healthcare Technology Hub, Ulster University, Jordanstown Shore Road, Northern Ireland BT37 0QB, United Kingdom; ⊥ Department of Chemistry, University of Bari Aldo Moro, Edoardo Orabona 4, Bari 70125, Italy; # Bari unit, Center for Colloid and Surface Science (CSGI), via della Lastruccia 3, Sesto Fiorentino 50019, Italy

**Keywords:** SAXS, spike proteins, rheology, viscosity, viruses

## Abstract

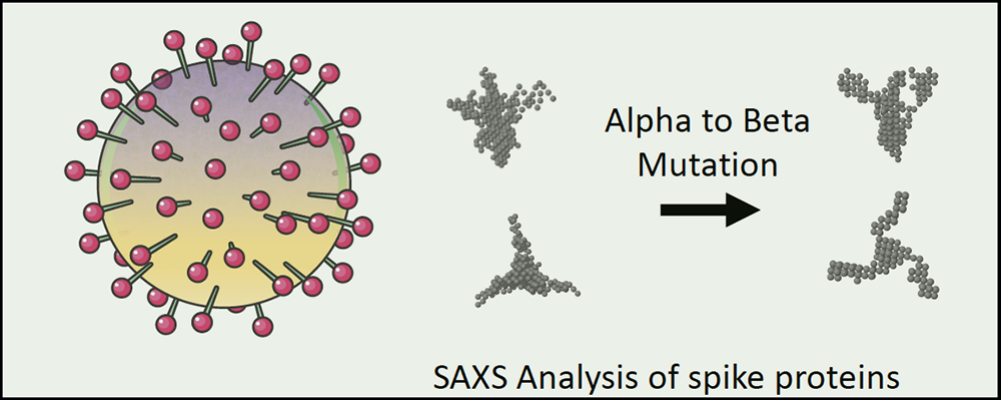

Measuring structural features of proteins dispersed in buffer solution, in contrast to crystal form, is indispensable in understanding morphological characteristics of the biomolecule in a native environment. We report on the structure and apparent viscosity of unfolded α and β variants of SARS-CoV-2 spike proteins dispersed in buffer solutions. The radius of gyration of the β variant is found to be larger than that of the α variant, while the ab initio computation of one of the possible particle-like bodies is consistent with the small-angle X-ray scattering (SAXS) profiles resembling a conformation similar to the three-dimensional structure of the folded state of the corresponding α and β spike variant. However, a smaller radius of gyration with respect to the predicted folded state of 2.4 and 2.7 is observed for both α and β variants, respectively. Our work complements the structural characterization of spike proteins using cryo-electron microscopy techniques. The measurement/analysis discussed here might be useful for quick and cost-effective evaluation of several protein structures, let alone mutated viral proteins, which is useful for drug discovery/development applications.

Small-angle X-ray scattering (SAXS) provides shape and supramolecular structural features of aggregates and large molecules in solution.^1,2^ SAXS can also act as an analytical method when data is reported on the absolute scale, e.g., providing a volume fraction estimation. For protein science, SAXS provides low-resolution information on protein shape, conformation, and the state of its assembly.^3^ However, SAXS measurements are relatively fast, and they also offer possible quantitative analysis of flexible systems such as those involving the intrinsically disordered proteins. SAXS can provide complementary information with respect to spectroscopy-based methods^4,5^ on the molecular state of aggregation in a reasonable experimental time depending on the X-ray source (from seconds to hours). SAXS has been used in the recent past to identify new structural insights in several protein interactions, which include human NLRP^6^ and kinases,^7^ to study the influence of heavy metals on the protein structure,^8^ to uncover the self-assembly mechanisms in proteins from a structural perspective,^9^ and even to investigate the interaction between amyloid-forming peptides and phospholipids.^10^ Such structural characterization of proteins is important in applications related to drug development and drug discovery processes for molecules. For instance, prior to the advent of X-ray-based techniques, such as SAXS, to identify three-dimensional (3D) structures of molecules, the inhibitory drugs were mostly discovered by systematic modification of known compounds that were discovered primarily via trial and error methods.^11^ Additionally, in structure-based drug design, computational approaches (so-called docking) with SAXS profiles can provide binding sites and affinities of molecules with their target.^12,13^ Recently, significant efforts have been put into understanding the structure of SARS-CoV-2 spike protein.^14−16^ Spike protein is a structural transmembrane glycoprotein of the SARS-CoV-2 virus and is involved in both receptor recognition and the cell membrane fusion process.^17^ It consists of two subunits, S1 and S2. The S1 subunit contains the so-called receptor-binding domain (RBD), the portion of the protein recognizing and interacting with the host receptor angiotensin-converting enzyme 2 (ACE2), while the S2 subunit is involved in viral cell membrane fusion. Therefore, investigating the structural features of the S protein is crucial to understanding the infection mechanism^18^ and developing SARS-CoV-2-specific drugs.^19^ The importance of aforementioned research theme and the rise of new SARS-CoV-2 mutants motivated us to perform SAXS characterization of 2 variants of the S protein (B.1.1.7, α and B.1.351, β).

In the SAXS experiment, the protein solution is exposed to X-rays, and the coherent and diffuse scattering at small angles from the incident beam is recorded on a two-dimensional (2D) area detector. The isotropic 2D pattern is averaged azimuthally leading to the one-dimensional (1D) SAXS profile where the scattering intensity, *I*(*q*), is plotted as a function of the scattering vector, *q* = (4π sin(θ/2))/λ, where θ is the scattering angle and λ = 1.54 nm is the X-ray wavelength. SAXS analysis provides information regarding the global molecular shape of the protein.^20^

Figure 1 shows the SAXS profile for the spike α solution at 0.3 mg/mL in HEPES buffer (10 mM) and MgCl_2_ (1 μM) at pH 7.5. To obtain the gyration radius, *R*_g_, without assuming any shape, we can adopt the classical Guinier analysis,^21^ ln(*I*(*q*)) vs *q*^*2*^ (Figure 1A inset), leading to *R*_g_ of 2.0 ± 0.1 nm with respect to the *q*-range considered (*qR*_g_ < 1.1). The Guinier approximation is given in eq 1:
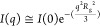
1where *I*(0) is the forward scattering intensity and *q* is the scattering vector magnitude. The Kratky plot, *I*(*q*)*q*^2^ vs *q*, provides the protein folding state in solution;^22^ in our case, a partially unfolded state is reported for spike α.

**Figure 1:**
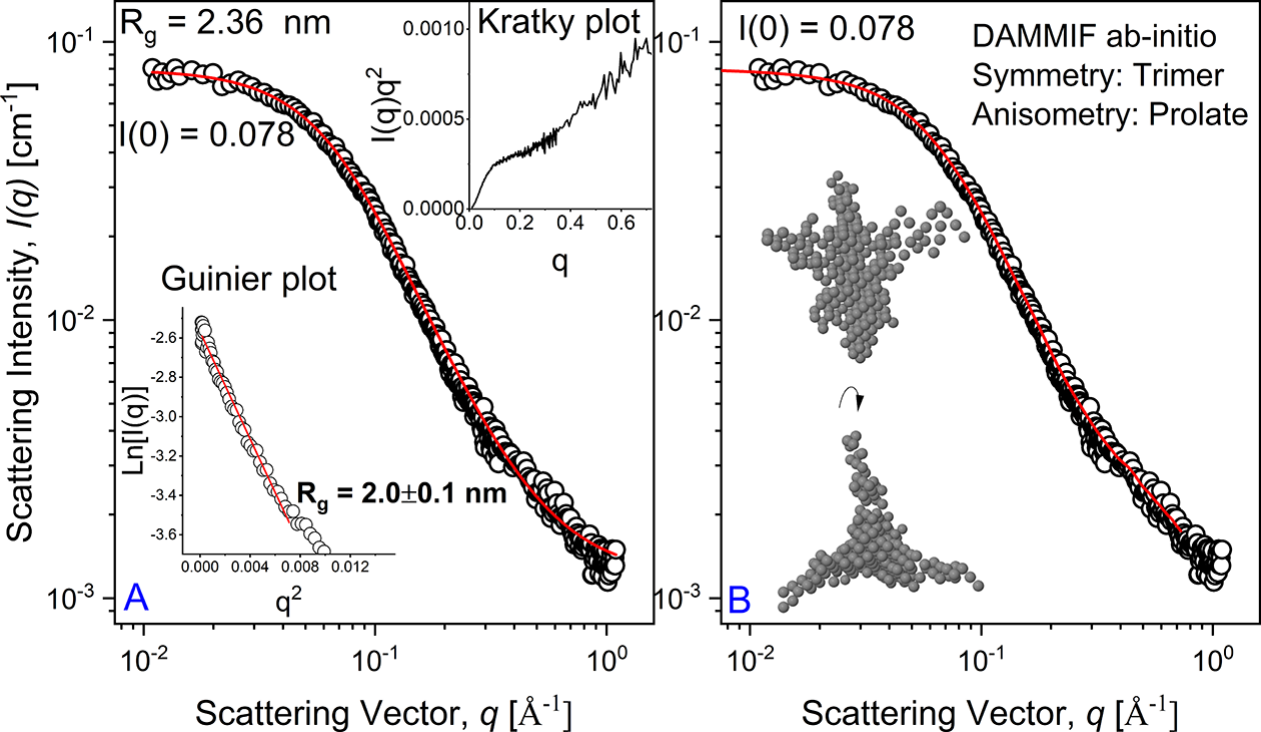
Small-angle X-ray scattering profile of the spike protein of the α variant of SARS-CoV-2 modeled using the Debye–Gaussian coil model (A) and the ab initio shape-determination by DAMMIF (B). Insets in panel (A) are the Guinier plot and the Kratky plot at the bottom and upper part, respectively. Inset in panel (B) is the result of the ab initio simulation of DAMMIF.

The Debye–Gaussian-distributed random coil has been the dominant model for denatured proteins in the past.^23^ Indeed, to a good approximation, end-to-end distances for random coils of sufficient length are Gaussian distributed,^24^ as confirmed by simulations on unfolded proteins.^25^ The Debye–Gaussian model, eq 2 (Figure 1A), appropriately models the scattering data, providing a *R*_g_ of ∼2.4 nm (relatively close to the Guinier approximation).
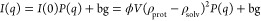
2where φ is the volume fraction of the protein in solution, ρ_prot_ is the scattering length density of the protein, ρ_solv_ is the scattering length density of the solvent, *V* is the volume of the protein coil equal to *M*_w_/(*N*_a_δ) (*M*_w_ is the molecular weight, *N*_a_ is Avogadro’s number, and δ is the bulk density of the protein), bg is the background, and *P*(*q*) is the form factor as described by eq 3.
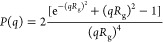
3The *R*_g_ value obtained is in agreement with simulations on similar spike proteins in the presence of glycyrrhizic acid.^26^ However, the FoXS (Fast X-ray Scattering)^27,28^ application was adopted to compute SAXS profiles on the basis of the 3D shapes of the crystalline proteins reported in the Protein Data Bank (PDB).^29^ The α variant B.1.1.7 was computed from the PDB protein code 7LWV, while the β variant B.1.351 was computed from the PDB protein code 7LYN. The protein α (PDB ID: 7LWV) leads to an *R*_g_ of 4.97 nm (see Figure 3). This is due to the unfolded state of the protein analyzed here, while the simulated crystalline state is clearly in the folded state.

The Debye–Gaussian-distributed random coil is a simple mathematical model from which many conformational properties can be derived. To provide a possible 3D conformation based on its SAXS profile, ab initio methods can be adopted to propose coarse shapes represented by dummy beads that fit the experimental profile.^30−32^ Ab initio methods reconstruct the low-resolution shape of proteins by modeling a small-angle scattering experimental profile.^33,34^ Here the DAMMIF algorithm^34^ has been adopted to determine the protein shape in solution (Figure 1). The prolate *C*3 symmetry has been considered the best choice considering the fitting residuals, even though the ab initio computation without symmetry assumption leads to similar results; see the Supporting Information. Assuming a trimer symmetry and a prolate anisometry, the obtained result matches the expected spike protein shape. The forward scattering intensity *I*(0) estimated at zero angle (*q* = 0) on an absolute scale is proportional to the molecular mass. *I*(*0*) obtained using eq 2 or from the ab initio simulation leads to the same *I*(0)_Debye–Gaussian_ = *I*(0)_ab-initio_ = 0.078 cm^–1^. The resulting φ is 1.2 × 10^–6^ considering the bulk density of the protein, δ = 1.35 g/cm^3^, ρ_prot_ = 11.6 × 10^–6^ Å^–2^, and molecular weight = 142 114 Da. The shape of the protein reported in Figure 1 is similar to shapes reported for membrane proteins of viruses.^35^Figure 2 reports the SAXS profile for the spike β protein solution at 0.25 mg/mL in the same buffer conditions of the spike α solution. In this case, the Debye–Gaussian approximation is not describing the SAXS profile properly and the radius of gyration obtained by the Guinier approximation is slightly larger than the one observed for spike α, equal to 2.7 ± 0.1 nm adopting the same *q*-range considered for spike α. A smaller *q*-range will lead to a maximum value of 3.2 ± 0.1 nm. The Kratky plot indicates a completely unfolded state. The diverging results between the α and β spike variants cannot be assigned only to the different concentration in buffer, while they might arise from a different conformation of the random coil. The simulated crystalline structure (PDB ID: 7LYN) leads to 4.94 nm (see Figure 3), which diverges from our experimental data due to the unfolded state of the protein.

**Figure 2:**
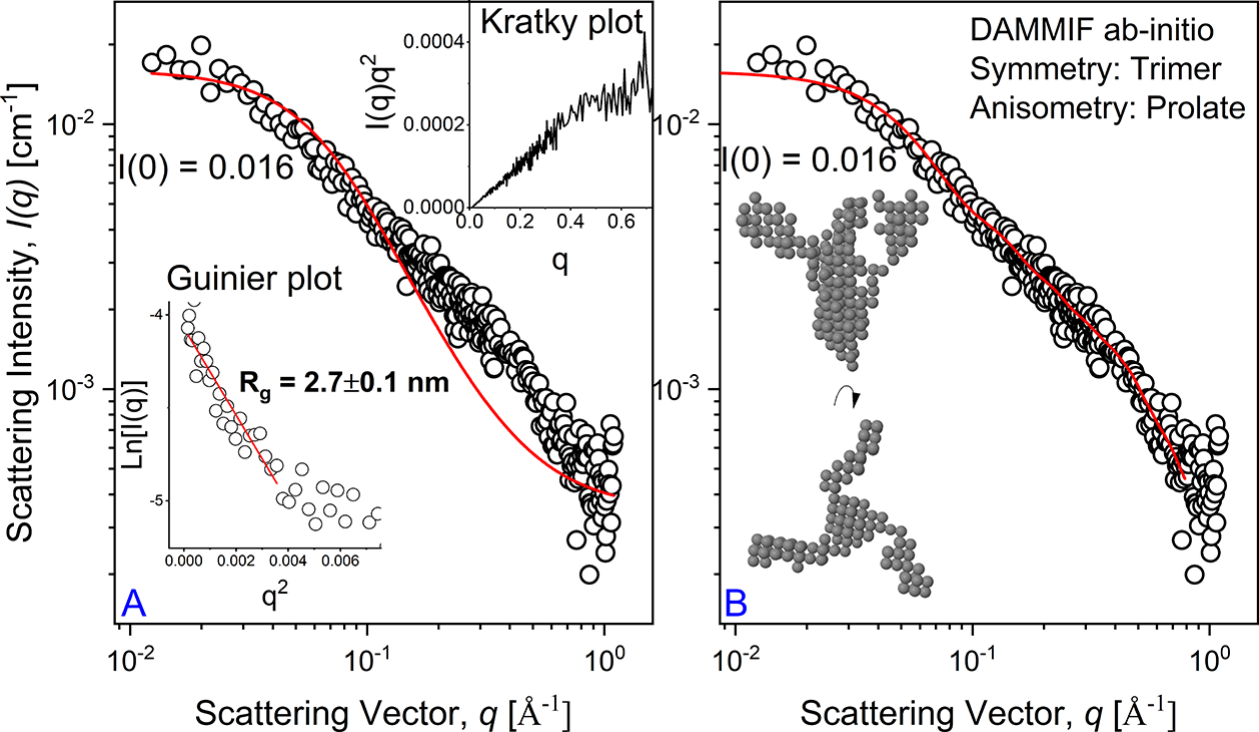
Small-angle X-ray scattering profile of the spike protein of β variant of SARS-CoV-2 modeled using the Debye–Gaussian coil model (A) and the ab initio shape determination by DAMMIF (B). Insets in panel (A) are the Guinier plot and the Kratky plot at the bottom and upper part, respectively. Inset in panel (B) is the result of the ab initio simulation of DAMMIF.

**Figure 3:**
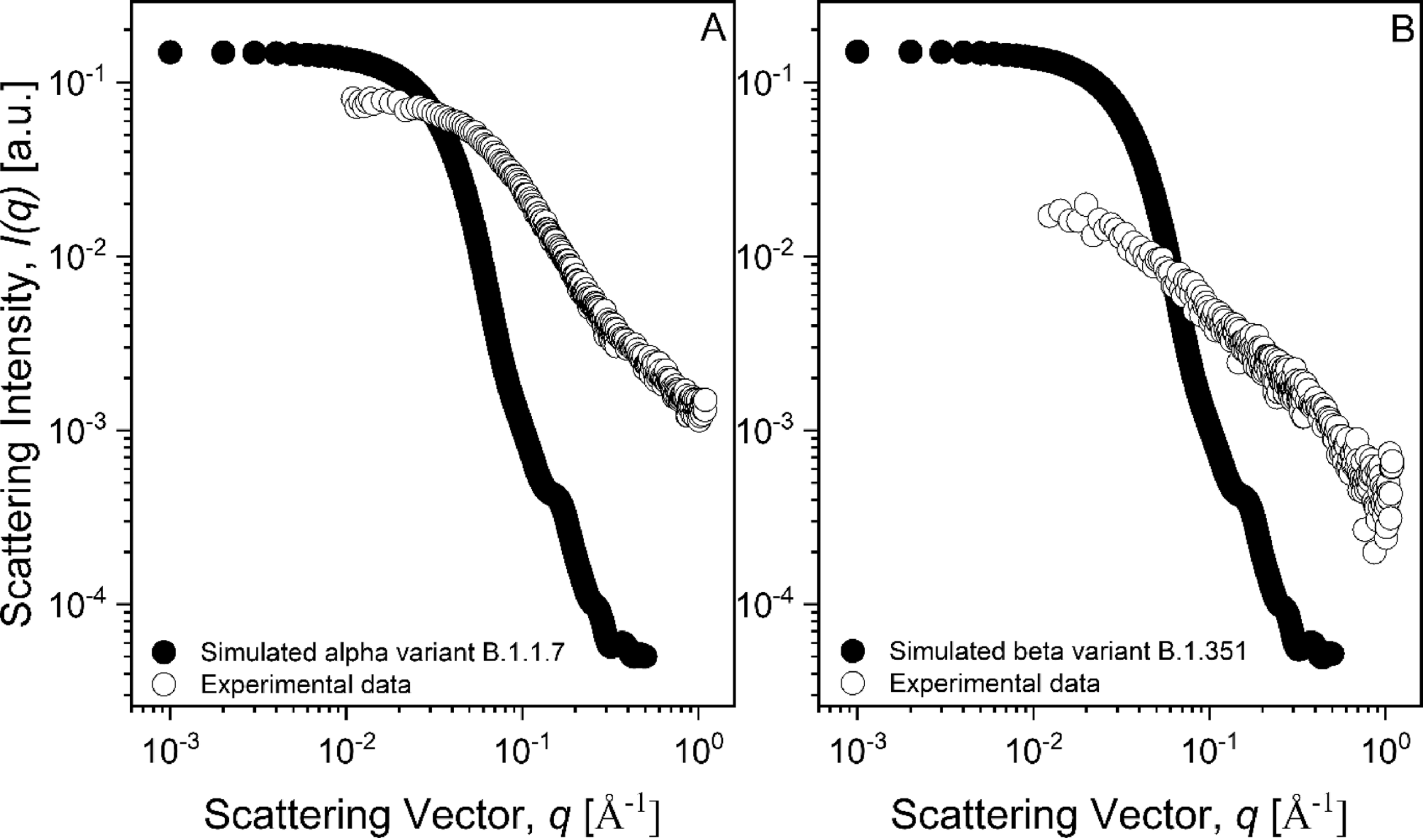
Experimental SAXS profiles (empty circles) and computed SAXS profiles (filled circles) for the α variant B.1.1.7 (A) and the β variant B.1.351 (B). The scattering intensity of the computed profiles has been arbitrarily scaled since the volume fraction was not taken into account.

The ab initio shape simulation reveals a more “open structure” with respect to the spike α. *I*(0) obtained using eq 2 or from the ab initio simulation leads to the same *I*(0)_Debye–Gaussian_*= I*(0)_*ab-initio*_ = 0.016 cm^–1^ for the spike β protein. The resulting φ is 2.4 × 10^–7^ considering δ = 1.35 g/cm^3^, ρ_prot_ = 11.6 × 10^–6^ Å^–2^, and molecular weight = 142 114 Da. The obtained volume fraction is more than 7 times smaller than the spike β protein concentration, while it was less than 2 times in the case of the spike α protein solution. The volume fraction φ is given by the product of the number of protein units in a given assembly and the dimensionless amount of that particular assembly per unit volume. The spike α protein solution has a radius of gyration smaller than that of the spike β protein; i.e., a higher fraction of molecules is assembled in the spike α protein solution. Future experiments on spike proteins by SAXS will be useful to confirm our initial results and to reveal further details. Generally, at relatively high concentrations, the interplay between the attractive van der Waals interactions and long-range electrostatic repulsive interaction forces is common for some proteins, including antibodies, and it governs clustering, phase behavior, and solution viscosity. The local concentrations of protein molecules clustered together, sometimes forming interconnected percolated filamentous networks, lead to solutions that are more viscous and sometimes to shear-thinning.^36−38^ On the contrary, here, the flow curves of the protein solutions reveal a Newtonian behavior^39^ for all investigated samples in the range 20–100 s^–1^ (Figure 4) excluding the clustering effect at the investigated concentration. In order to provide reliable data, water was measured in the same experimental condition as a reference value. Further investigation as a function of the concentration will provide the intrinsic viscosity.

**Figure 4:**
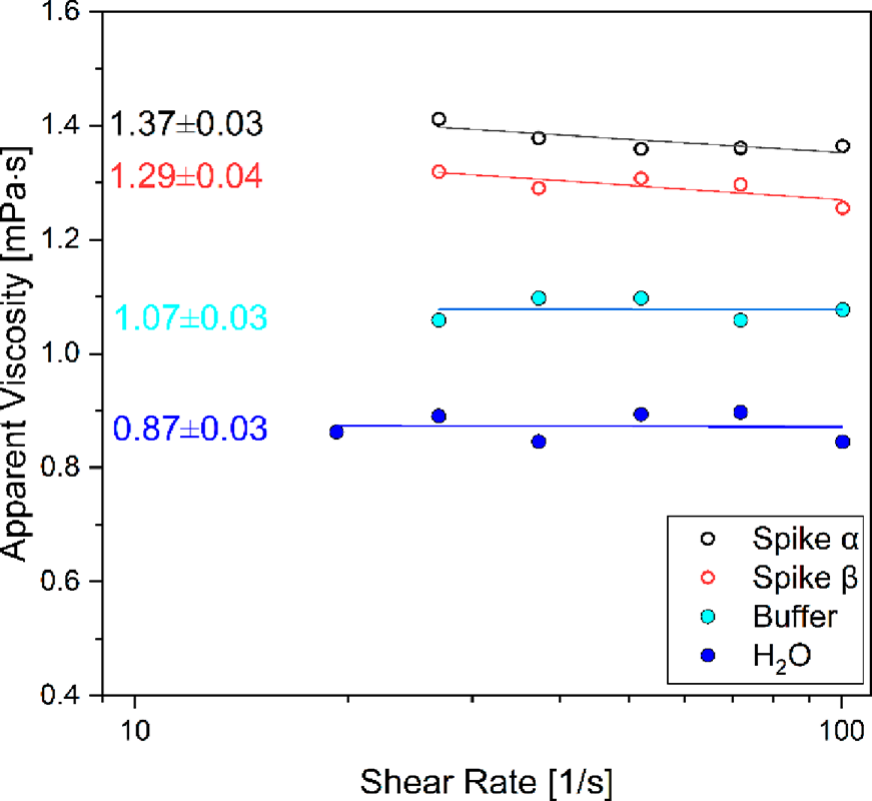
Viscosity profiles measured for the proteins of α, β variants of SARS-CoV-2 in Hepes + MgCl_2_ buffer along with the buffer and H_2_O viscosity profiles and the zero-shear viscosity obtained by linear fitting.

We show for the first time the use of SAXS for quick structural analysis of those proteins which belong to the evolving viral variants. We show using ab initio simulation that upon the evolution of the virus to a new strain, the spike proteins in the buffer show a random-coil conformation resembling the 3D structure of the folded proteins. In other words, the random coil conformation might resemble the 3D structure of the folded protein. In addition, the change in radius can also result from K417N mutation which is one of the main differences between α and β variants.

Finally, the Newtonian flow curve has proven that there is no formation of clustering at the investigated concentration. Along similar lines, proteins from other variants of SARS-CoV-2 (or other potential pandemic strains) can be analyzed to reveal structural information in a short span of time in buffer solutions that might be useful for drug and vaccine development in a short time. Our study also complements several other works in which utilize viral proteins in similar buffers for bioassays as we show that proteins retain their shape and structure in buffer conditions.^40,41^ SAXS could also provide new knowledge in an uncertain area of understanding how protein structures in viruses evolve when a virus mutates. Therefore, our work demonstrates a benchmark for an easy and fast structural analysis of evolving variants of virus proteins.

## Methods

α (ABIN6963742) and β (ABIN6963740) variants of the SARS-CoV-2 spike protein were purchased from antibodies-online.com. Both are recombinant proteins produced in human cells (HEK-293). The two variants differ from the canonical sequence of the spike protein as follows. Del 69-70, del 144, N501Y, A570D, D614G, P681H, T716I, S982A, and D1118H are mutations characteristic for the SARS-CoV-2 α variant (B.1.1.7), while del 144, K417N, E484 K, N501Y, A570D, D614G, P681H, T716I, S982A, and D1118H mutations are indicative for the SARS-CoV-2 β variant (B.1.351). The molecular weight of the proteins (142 114 Da), UniProt ID (P0DTC2), SDS PAGE analysis, and purification information (purity > 98%) are also provided by the manufacturer. The purified α spike protein solution having a concentration of 0.3 mg/mL in HEPES (10 mM) + MgCl_2_ (1 μM) buffer at the resulting pH of 7.5 was investigated by means of SAXS. Note that these are the handling conditions suggested by the manufacturer; thus, we use the aforementioned buffer conditions to analyze the protein in solution.^40^

The spike proteins, buffer, and water flow curves were carried put using the MCR302e stress-controlled rheometer (Anton Paar Gmbh, Graz, Austria) equipped with a cone–plate geometry with a measuring tool diameter of 25 mm and an angle of 1°. The temperature was kept to 25 °C by a Peltier system. The difference in viscosities between the proteins is not so significant since their value is at the instrumental edge limits. However, the presence of aggregates would lead to a small shear thinning since those aggregates would disentangle to align in the flow direction.

SAXS measurements were performed using a pinhole-collimated system equipped with a Genix 3D X-ray source (Xenocs SA, Sassenage, France) produced by SAXSLAB ApS, Skovlunde, Denmark. The scattering intensity, *I*(*q*), was recorded with the Pilatus detector (Dectris Ltd., Baden, Switzerland) located at two distinct distances from the sample, providing a scattering vector range 0.0042 Å^–1^ ≤ *q* ≤ 1.00 Å^–1^, even though the spike protein scattering intensity was detected only from 0.012 Å^–1^. The spike proteins were loaded in a 1.5 mm diameter quartz capillary and then sealed (Hilgenberg GmbH, Malsfeld, Germany). An external JULABO thermostat (JULABO, Seelbach, Germany) fixed to 25 °C controlled the temperature. The two-dimensional (2D) scattering pattern was radially averaged using SAXSGui v2.15.01 software to obtain *I*(*q*). The measured scattering curves were corrected for the background scattering.

The SAXS data analysis was conducted with both a classical modeling approach and by using ab initio computation. The ab initio computation is a useful way to analyze the intricate scattering profiles of proteins in solution. In fact, the random motion and orientation of molecules lead to a loss of spatial orientation information due to an effective averaging of the scattering. To overcome this issue, we can assume a 3D model of the protein backbone by using ab initio computation in the analysis.^42^

## Supporting Information Available

Ab initio computation of the scattering profile without symmetry assumption; residuals of the adopted models (PDF)
