# Supplementary material for: Measuring the Radius of Gyration and Intrinsic Flexibility of Viral Proteins in Buffer Solution Using Small-Angle X-ray Scattering
Source: ACS Meas Sci Au. 2022 Sep 12;2(6):547–52. doi: 10.1021/acsmeasuresciau.2c00048 (PMC9783065; doi:10.1021/acsmeasuresciau.2c00048)
Supplement: Supplementary file 1 — tg2c00048_si_001.pdf [file tg2c00048_si_001.pdf]

Measuring the radius of gyration and intrinsic flexibility of viral proteins in buffer solution using small angle X-ray scattering

Riccardo Funari [a,b], Nikhil Bhalla*[c,d] and Luigi Gentile*[e,f]

[a] Department of Physics "M. Merlin", University of Bari Aldo Moro, Via Amendola, 173, Bari, 70125, Italy.

[b] Institute for photonics and nanotechnologies, CNR, Via Amendola, 173, Bari, 70125, Italy

[c] Nanotechnology and Integrated Bioengineering Centre (NIBEC), School of Engineering, Ulster University, Jordanstown, Shore Road, Northern Ireland, BT37 0QB, United Kingdom, email: n.bhalla@ulster.ac.uk

[d] Healthcare Technology Hub, Ulster University, Jordanstown Shore Road, Northern Ireland, BT37 0QB, United Kingdom

[e] Department of Chemistry, University of Bari Aldo Moro, Edoardo Orabona 4, Bari, 70125, Italy, email: luigi.gentile@uniba.it

[f] Bari unit, Center for Colloid and Surface Science (CSGI), via della Lastruccia 3, Sesto Fiorentino, 50019, Italy.

Simulation comparison

In the main text, the DAMMIF algorithm [1] has been adopted to determine the protein shape in solution assuming a trimer symmetry and a prolate anisometry. Here in comparison, the DAMMIF algorithm [1] has been adopted without symmetry assumption for both α and β spike variants, Figure S1. The computed profile is very similar to the one suggested in the main text.

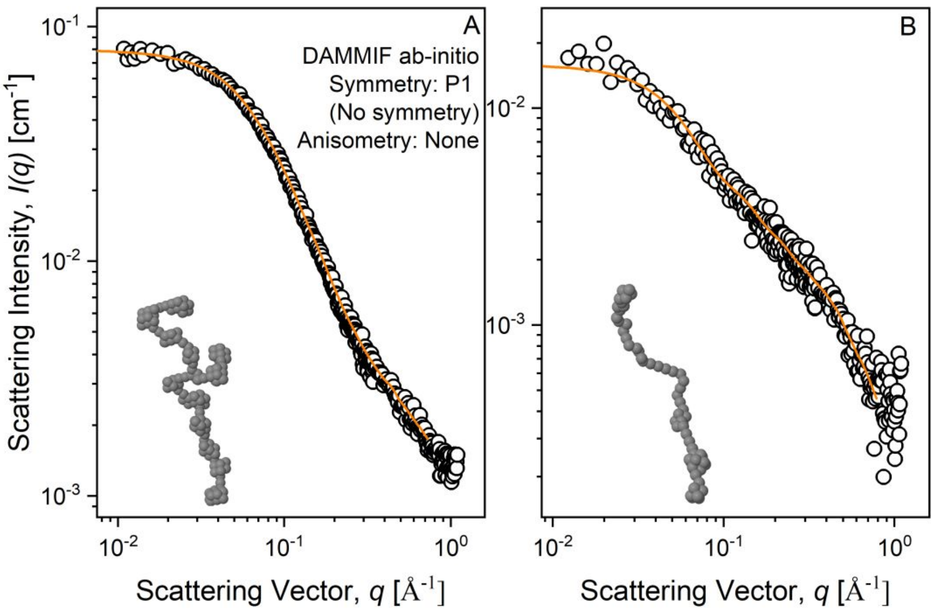

Figure S1. *Figure. 1 Small-angle X-ray scattering profile of the spike protein of α (A) and β (B) variants of SARS-CoV-2 and the ab-initio shape determination by DAMMIF assuming no symmetry (P1). The figure caption on the panela A and B are the result of the ab-initio simulation of DAMMIF.*

The residuals of the Debye-Gaussian coil, the ab-initio computation without symmetry (P1), and the ab-initio computation considering prolate C3 symmetry are reported for the α and β spike variants in Figure S2 and Figure S3, respectively. It can be appreciated clearly, specifically for the β variant, that the Debye-Gaussian coil is diverging from 0 more than the other two. On the other hand, the computed ab-initio models are very close to each other, even though the residuals of the one considering the prolate C3 symmetry are closer to 0.

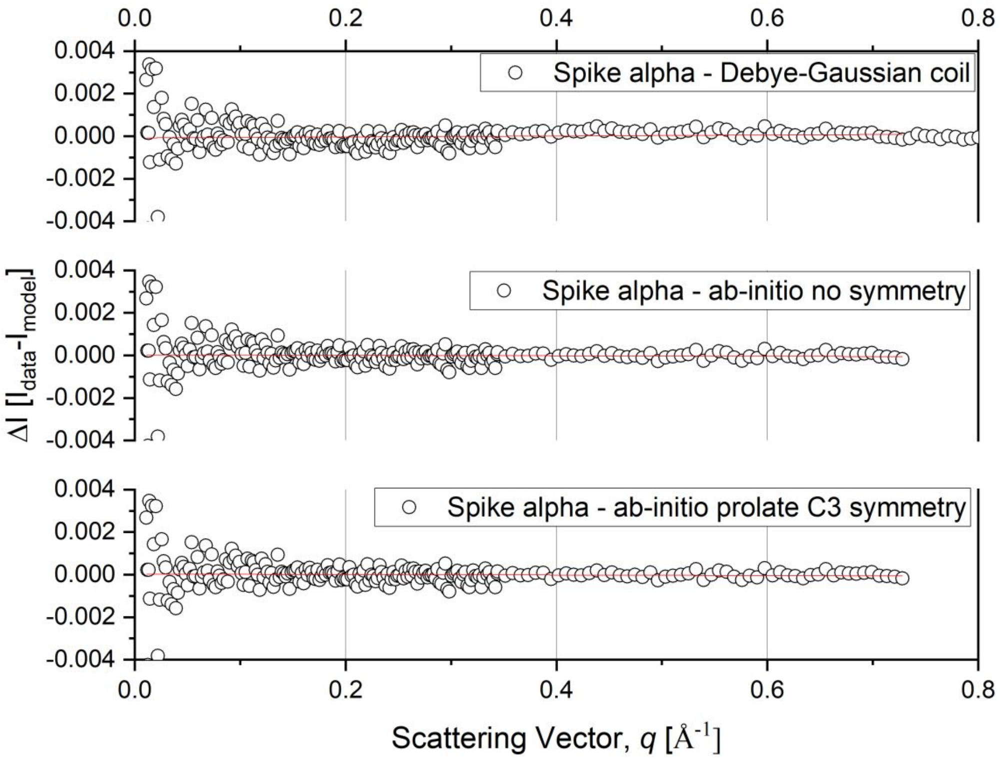

Figure S2. *Residuals of the Debye-Gaussian coil, the ab-initio computation with no symmetry assumption and the ab-initio computation with prolate C3 symmetry for the spike α variant.*

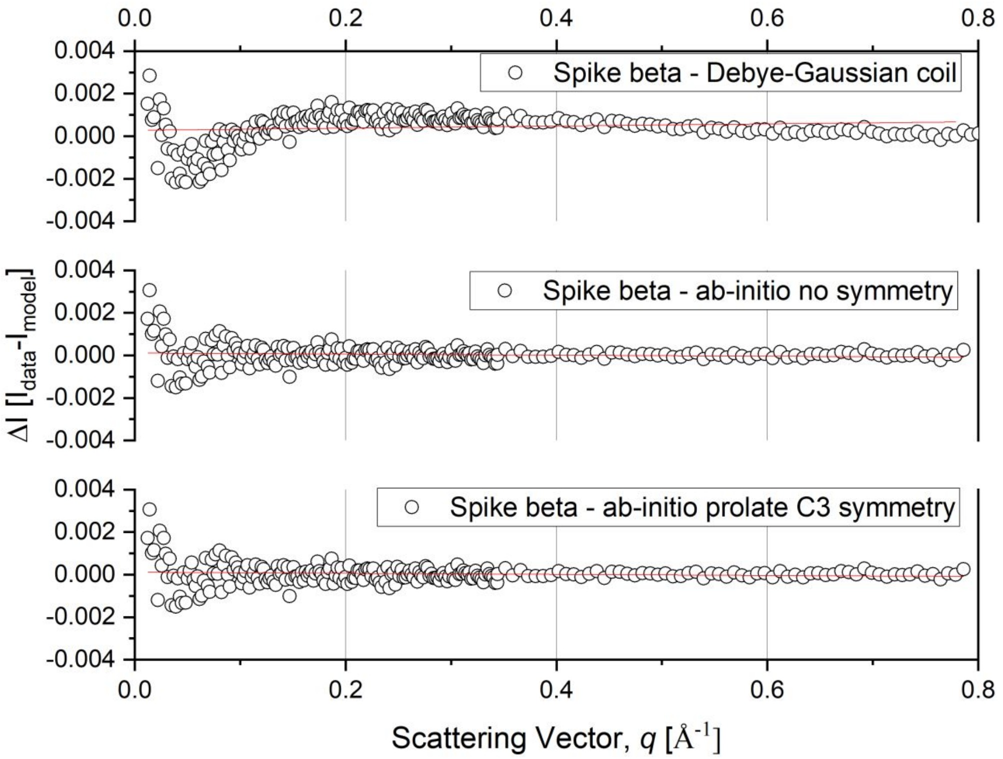

Figure S3. *Residuals of the Debye-Gaussian coil, the ab-initio computation with no symmetry assumption and the ab-initio computation with prolate C3 symmetry for the spike β variant.*
